# Sarcoidosis Manifesting as Liver Granuloma With Asteroid Bodies

**DOI:** 10.7759/cureus.17915

**Published:** 2021-09-12

**Authors:** Adel Muhanna, Laith Al Momani, Alisa Likhitsup

**Affiliations:** 1 Internal Medicine, University of Missouri Kansas City School of Medicine, Kansas City, USA; 2 Gastroenterology, University of Missouri Kansas City, Kansas City, USA; 3 Gastroenterology and Hepatology, University of Missouri Kansas City, Kansas City, USA

**Keywords:** asteroid bodies, sarcoidosis, liver granuloma, elevated alkaline phosphatase, autoimmune disorder

## Abstract

Sarcoidosis is an autoimmune disease, which most commonly affects the lungs and lymph nodes and is characterized with non-caseating granulomas. Hepatic involvement in sarcoidosis occurs in less than 1% of patients. Most patients with hepatic sarcoidosis remain asymptomatic with only laboratory abnormalities. We present the case of a 59-year-old man with sarcoidosis who was evaluated for an elevation of alkaline phosphatase. Laboratory test results revealed an alkaline phosphatase level of 230 U/L, with normal alanine aminotransferase, aspartate aminotransferase, bilirubin, and albumin. Computed tomography of the abdomen and pelvis with intravenous contrast of the liver showed a mildly enlarged liver. Liver biopsy sections showed steatosis, active steatohepatitis, and focal portal granuloma formation with asteroid body. The patient was scheduled regular liver function tests and clinical monitoring. Most patients with hepatic sarcoidosis remain asymptomatic with only laboratory abnormalities such as elevation of liver enzymes and alkaline phosphatase. Although liver involvement is common in gastrointestinal sarcoidosis, progression to liver cirrhosis is rare in such patients. While symptomatic patients may be managed with systematic prednisone, asymptomatic patients may require only laboratory and clinical monitoring.

## Introduction

Sarcoidosis is an autoimmune disease, which most commonly affects the lungs and lymph nodes and is characterized with non-caseating granulomas. It has an estimated prevalence of 10 cases per 100,000 population worldwide [1]. Gastrointestinal tract involvement in sarcoidosis is present in less than 1% of cases with the stomach being the most common organ involved [2], and hepatic involvement is more common. Approximately, 50-60% of patients with gastrointestinal sarcoidosis have granulomas on liver biopsy; however, symptomatic hepatic sarcoid occurs in 5-15% of cases [3-5]. Herein, we present the case of a 59-year-old man with sarcoidosis presenting with abnormal liver function tests. 

## Case presentation

**Figure 1:**
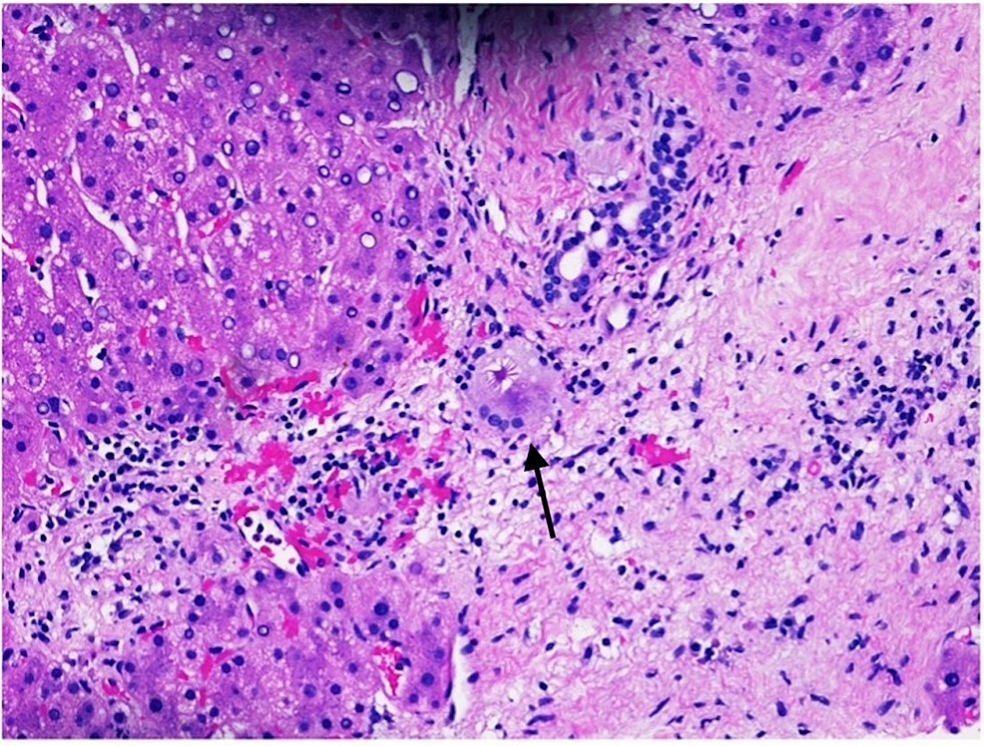
Hematoxylin and eosin stain of liver biopsy (20x). Liver biopsy showing characteristic asteroid bodies (black arrow).

**Figure 2:**
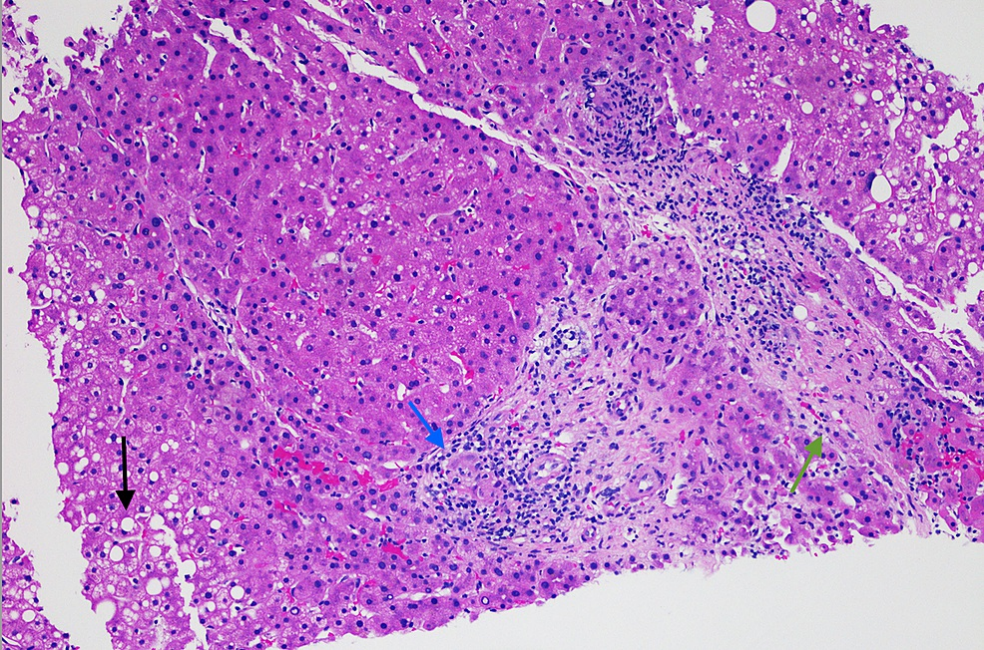
Hematoxylin and eosin stain of liver biopsy (20x). Liver biopsy showing active steatohepatitis (black arrow), focal portal granuloma formation (blue arrow), and increased bridging fibrosis (green arrow).

A 59-year-old man was evaluated for an elevation of alkaline phosphatase in the clinic. The patient has a past medical history significant for diabetes mellitus, sarcoidosis, cardiomyopathy, non-Hodgkin’s lymphoma, and hypertension. The patient is a non-smoker, and he denied any significant alcohol or drug use or known liver disease in himself or his family members. Upon physical examination, the patient was well appearing, he had no apparent jaundice, abdominal pain, and was in no apparent distress. Laboratory data revealed a hemoglobin of 11.6 g/dL, white blood cell count of 12 k/μL, and a platelet count of 535 k/μL. Other liver enzyme levels were normal with an aspartate aminotransferase level of 27 U/L, alanine aminotransferase levels of 29 U/L, an albumin level of 3.7 g/dL, and a total bilirubin level of 0.2 mg/dL. His alkaline phosphatase level was 230 U/L, and gamma-glutamyl transpeptidase level was 60 IU/L. Liver enzyme levels of the patient obtained two months ago were normal. The patient tested negative for viral hepatitis, antinuclear antibodies, anti-smooth muscle antibody, antimitochondrial antibody, and his alpha 1 antitrypsin and ceruloplasmin levels were normal. Computed tomography of the abdomen and pelvis with intravenous (IV) contrast of the liver showed a mildly enlarged liver with an extensive inhomogeneous uptake of fluorodeoxyglucose throughout the liver without discrete masses or nodules. Ultrasound-guided liver biopsy showed asteroid bodies (Figure 1) with active steatohepatitis, focal portal granuloma formation, and increased bridging fibrosis (Figure 2). Iron, Periodic acid-Schiff (PAS), and PAS-D staining of the liver were negative, and those findings were consistent with hepatic sarcoidosis. Following liver biopsy, the patient was scheduled regular liver enzymes for follow-up, and he was also scheduled repeat abdominal imaging in six months to monitor the liver closely. 

## Discussion

Sarcoidosis is a multisystem, autoimmune disease of unknown etiology characterized by the presence of non-caseating granulomas in the affected organs, with pulmonary involvement as the most common site of disease activity [1]. The most common finding of histopathology in sarcoidosis is the classic non-necrotizing granulomas with a central area of multinucleated giant cells, macrophages, CD4-positive T lymphocytes, and epithelioid cells [6]. A large, multicenter study on 700 patients with sarcoidosis and 30,000 relatives could not identify a genetic locus or an etiologic agent that was clearly implicated in the pathogenesis of sarcoidosis [7].

While pulmonary involvement is the most common, up to 30% of patients present with extrapulmonary involvement [8]. Skin and eye involvement occur in approximately 25% of patients, and are often early findings [9,10]. Gastrointestinal sarcoidosis occurs in 0.1-0.9% of patients [2], with the stomach being the most commonly involved portion of the gastrointestinal tract. Liver hepatic involvement occurs in about 12% of patients with sarcoidosis, which further adds to the rarity of our case. Patients with hepatic involvement can be asymptomatic and might have laboratory abnormalities at the time of presentation [11]. While the patient in our case presented with an asymptomatic elevation in alkaline phosphatase, some patients reported symptoms of abdominal pain, pruritus, and jaundice [4]. Hepatic sarcoidosis usually causes increased aminotransferases in 50-70% of cases with a less degree of elevation in serum alkaline phosphatase [4,12], unlike the normal findings of aminotransferases in our case. Rarely, hyperbilirubinemia and hypoalbuminemia might be present in cases of severe hepatic sarcoidosis causing liver cirrhosis [13]. 

Computed tomography of the abdomen and pelvis with IV contrast of the liver typically shows hepatomegaly and hypodense nodular lesions that can vary in sizes [14]. However, there were no masses on imaging observed in the case of our patient, and mild hepatomegaly was noted. Liver biopsy is usually recommended in cases of liver function test abnormalities [15]. Non-caseating granulomas are the most common histopathologic finding in hepatic sarcoidosis [11], but asteroid bodies are only found in about 10% of cases [16], as in the case of our patient. The prognosis in hepatic sarcoidosis is generally favorable, with less than 1% of cases in patients with sarcoidosis progressing to liver cirrhosis and portal hypertension, cholestatic liver disease, hepatic vein thrombosis, and sclerosing cholangitis [11]. The decision to treat gastrointestinal sarcoidosis depends on the severity and activity of the disease. In general, asymptomatic patients with mild elevation in laboratory chemistries do not require treatment and can be managed with close and regular follow-ups [17].

## Conclusions

In this case presentation, we reported the case of a patient with known sarcoidosis who presented with an elevation of his alkaline phosphatase as a manifestation of hepatic sarcoidosis. The treatment of hepatic sarcoidosis is still not well defined. Although liver involvement is common in gastrointestinal sarcoidosis, end-stage liver disease is a rare complication in such patients. Asymptomatic hepatic sarcoidosis can be monitored with repeat laboratory testing including transaminase and alkaline phosphatase levels.
